# Regional Nasal Drug Deposition in Pediatric vs. Adult Models: *In vitro* Insights into User Technique and Breathing Patterns Sensitivity

**DOI:** 10.1007/s11095-026-04066-8

**Published:** 2026-03-16

**Authors:** Mohammad Hejazi, Xiomara Owen, David J. Edwards, Michael Hindle, Worth Longest, Theodore Schuman, Ross Walenga, Steven Chopski, Anubhav Kaviratna, Bryan Newman, Laleh Golshahi

**Affiliations:** 1 Department of Mechanical and Nuclear Engineering, Virginia Commonwealth University, Room 4326, 401 W Main St, Richmond, VA 23220 USA; 2 Department of Statistical Sciences and Operations Research, Virginia Commonwealth University, Richmond, VA USA; 3 Department of Pharmaceutics, Virginia Commonwealth University, Richmond, VA USA; 4 Department of Otolaryngology - Head and Neck Surgery, VCU Health, Richmond, VA USA; 5 Division of Quantitative Methods and Modeling, Office of Research and Standards, Office of Generic Drugs, Center for Drug Evaluation and Research, U.S. Food and Drug Administration, Silver Spring, MD USA; 6 Division of Therapeutic Performance I, Office of Research and Standards, Office of Generic Drugs, Center for Drug Evaluation and Research, U.S. Food and Drug Administration, Silver Spring, MD USA; 7 Department of Biomedical Engineering, Virginia Commonwealth University, Richmond, VA USA

**Keywords:** nasal sprays administration parameters, predictive model, regional *in vitro* drug deposition, response surface methodology, targeted intranasal drug delivery

## Abstract

**Purpose:**

Although in vitro studies recommended for establishing bioequivalence of locally acting nasal suspension products provide useful information regarding product performance, currently they are not designed to fully account for variability introduced by patient-specific factors such as administration technique and breathing patterns. This limitation, combined with ethical and practical challenges in conducting pediatric clinical trials, creates a knowledge gap in understanding age-related differences in nasal drug delivery.

**Methods:**

Anatomically realistic in vitro nasal models representing an average child and adult, in terms of total drug delivery to the sites of intended action posterior to nasal valve, were used to investigate the sensitivity of regional nasal drug deposition to variations in administration parameters and breathing conditions. Three breathing conditions were evaluated: breath hold, gentle sniffing, and vigorous sniffing. Administration parameters were varied using a Box-Behnken experimental design.

**Results:**

Results showed that for commercially available nasal sprays, breathing patterns did not significantly affect drug delivery in the adult model, but extreme conditions (no breathing vs. vigorous sniffing) caused significant differences in anterior deposition in the child model. Differences between gentle sniffing and the other conditions were not statistically significant. The sensitivity analysis revealed that in vitro drug deposition patterns differ between child and adult nasal cavities under identical user parameter variations, and variable importance differed across nasal cavity regions.

**Conclusions:**

This study demonstrates the potential for targeted drug delivery by enabling the identification of the optimal administration parameters to achieve desired outcomes, such as enhanced drug deposition in specific nasal regions.

**Supplementary Information:**

The online version contains supplementary material available at 10.1007/s11095-026-04066-8.

## Introduction

Previous *in vitro* studies employing patient-specific spray administration conditions have demonstrated comparable *in vitro* drug deposition patterns in adults and pediatric subjects [1, 2]. However, in real-world settings, even if an optimized set of administration parameters could be identified, it would be challenging to ensure that patients consistently replicate the optimal configuration with each actuation of the nasal drug product. Literature reports have shown that most patients do not follow the current guidelines on spray use technique [3]. Moreover, these guidelines often lack detailed instructions regarding insertion depth and angles of spray administration [4]. Numerous studies, *in vivo* [5–7], *in vitro* [8–14], or in silico [15–21], have demonstrated that intranasal drug deposition pattern is heavily governed by administration parameters. Furthermore, the conclusions on the relative importance of each parameter across published studies remain contradictory, and has yet to be clearly established [22]. In addition, Hejazi *et al*. [13] showed that the most influential parameters affecting posterior drug deposition in children differ from those identified in adults [12]. This suggests that changing a given parameter in children may yield a different outcome compared to adults, particularly in terms of enhancing or reducing drug deposition in the turbinate region, which is a primary target site for most locally acting nasal drug products. Therefore, the observed comparability in deposition performance between children and adults may be limited when patient specific parameters are not implemented.

European Medicine Agency (EMA) and U. S. Food and Drug Administration (FDA) guidelines state that due to ethical considerations, children should not be included in clinical studies unless their participation is ethically justified and carefully regulated [23, 24]. Exceptions can be made when the developed drug is either addressing a major health need in pediatric population or there is small or no overlap between adult and pediatric condition. However, based on the current FDA guidelines [25–27], these exceptions are generally not made for suspension formulations of locally acting nasal drug products for pediatric population, and conclusions for bioequivalence as determined using these *in vitro* and *in vivo* studies is generally extended to the pediatric population via extrapolation [28]. To better understand the critical factors affecting performance in pediatric drug delivery, EMA and FDA suggest implementation of mathematical/statistical models and simulations to better predict age-specific outcomes [23, 24]. In this study, we coupled *in vitro* regional intranasal drug deposition, under different administration parameters and breathing conditions, with a mathematical/statistical model. *In vitro* regional drug deposition studies were carried out using pediatric and adult anatomical nasal models, and statistical modeling was done using response surface methodology (RSM). This study design enabled a comparative exploration of how regional drug deposition is governed by variations in spray administration and breathing conditions across pediatric and adult populations.

In addition to highlighting some of the challenges with developing *in vitro* regional drug deposition study methods that may be reflective of *in vivo* performance for different patient populations, this study offers valuable insights into targeted intranasal drug delivery. Beyond intranasal local drug delivery, this route of administration offers both established and emerging therapeutic benefits, including systemic drug delivery, vaccination, and nose-to-brain delivery, broadening its clinical application potentials. For systemic delivery, there are several nasal products that are used to treat migraine attacks in both adults and pediatrics [29–31], opioid overdose [32, 33], central cranial diabetes insipidus and nocturia [34, 35], and depression [36, 37]. Additionally, since most of the antigens start on nasal surface and mucosa [38], nasally-administered vaccines are also of interest for immunization against respiratory diseases. However, there is a limited number of intranasal vaccines that have been approved for use by FDA [22, 39]. Moreover, interest in nose-to-brain delivery has significantly increased, as it exhibits great potential to treat central nervous system diseases [40] such as neuroAIDS [41–43], Alzheimer’s disease [44, 45], and psychiatric disorders [46, 47]. To fully exploit these potential benefits of intranasal drug delivery, efficiently targeting the drug to the region of interest in nasal cavity is vital. In this study, we developed two predictive models, one for the pediatric and one for the adult nasal geometry, based on administration angles and insertion depth of a standard nasal spray pump. These models can be used either to optimize the drug delivery to any desired nasal regions, or to estimate the percentage of drug deposited in specific regions given a set of administration parameters.

## Methods

### Nasal Models

**Fig. 1:**
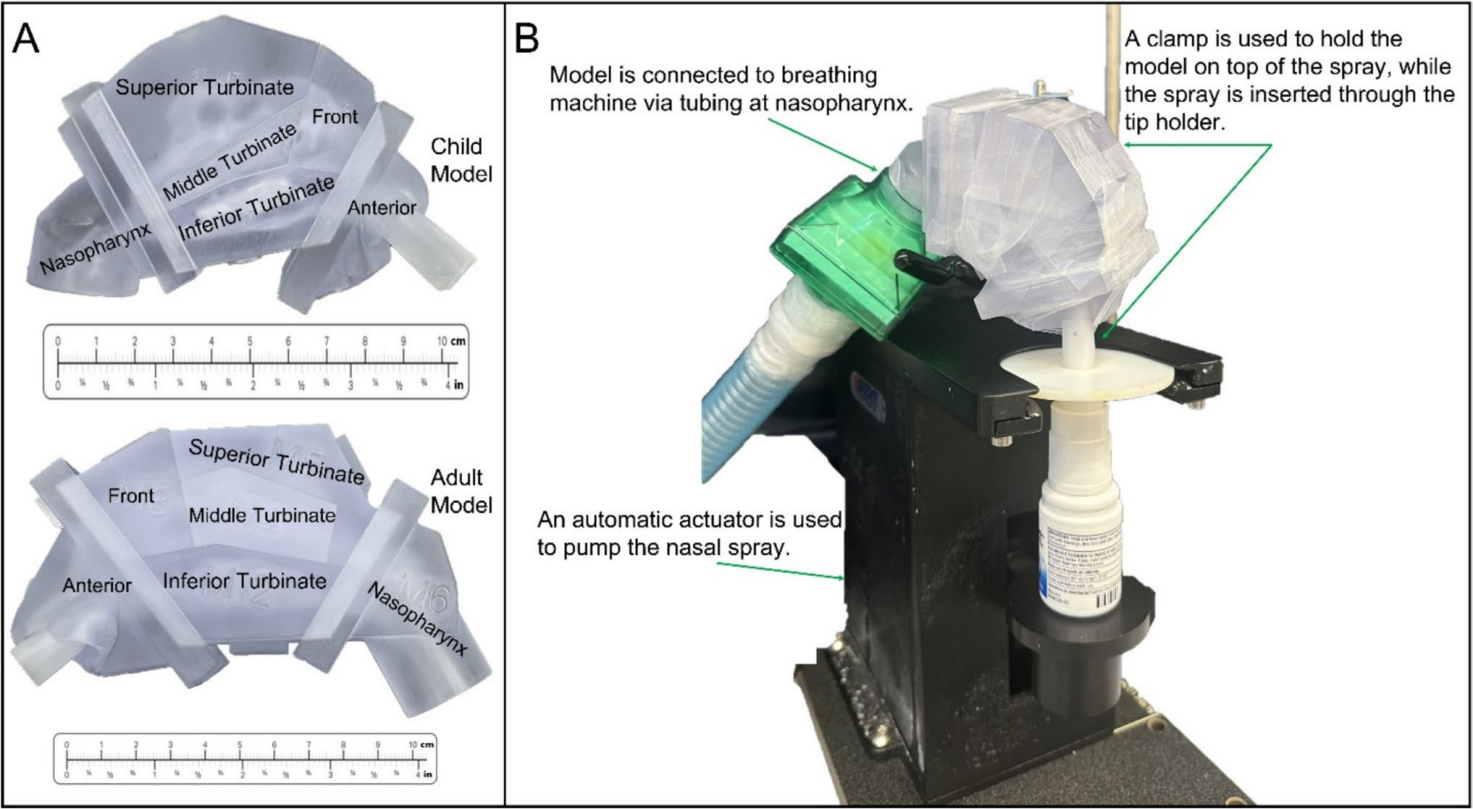
**A**) The child and the adult nasal models, and their segments, **B**) The experimental setup.

Our group has previously developed three pediatric and three adult CT-based anatomically realistic nasal models that capture intersubject variability within each population [2]. These models serve as representative geometries for evaluating drug delivery performance across a range of anatomical differences, with detailed consideration of location of internal nasal valve in each geometry [1, 48, 49]. The selection of these models was based on the percentage of drug deposition of two different nasal sprays in the posterior region of 40 healthy nasal cavities in each age group, to represent three levels of posterior deposition: low (L), mean (M), and high (H) [1, 50]. The M pediatric and adult models, representing the mean posterior deposition in their respective population, were used in this study. The child M model is based on a 5-year-old female subject, and the adult M model is based on a 35-year-old male subject. These models are segmented into anterior, front, inferior turbinate, middle turbinate, superior turbinate, and nasopharynx to C1 vertebrae (NC1), and allow us to examine *in vitro* regional drug deposition in the nasal cavity (Fig. 1A). Segmentation of the anterior piece was done by identifying the internal nasal valve (INV) plane [49], and subsequently the posterior region was segmented into five pieces. The process of segmentation of the nasal models is explained by Khadka *et al*. [2].

### Design of Experiments

In this study, we independently examined the effects of breathing patterns and administration parameters on *in vitro* regional drug deposition. The following sections provide detailed designs of experiments for each parameter.

#### Breathing Pattern Sensitivity Analysis

To investigate the effect of breathing patterns on regional drug deposition, three distinct conditions were evaluated: no breathing (breath hold), gentle sniff, and vigorous sniff following actuation (Table S1) [51]. In both gentle and vigorous sniff conditions, the flow rate increased approximately linearly from zero to the peak flow rate (PFR) over the time interval defined by T_max_, remained constant at the PFR for T_d_—T_max_ seconds, then decreased approximately linearly back to zero over the same duration T_max_ (Figure S1). Three units of Nasacort® Allergy 24HR (triamcinolone acetonide, 55 mcg per spray) nasal metered spray and three units of Flonase® Sensimist™ Allergy Relief Nasal Spray for 24HR (fluticasone furoate, 27.5 mcg per spray), all from the same lot, were used in the *in vitro* regional drug deposition experiments. To ensure a direct comparison between the two models, identical breathing patterns were used across both models to isolate the impact of nasal anatomy. This controlled approach allowed for a direct assessment of how anatomical differences between the child and the adult model modulate deposition sensitivity under equivalent flow conditions.

#### Sensitivity Analysis of Administration Parameters

To explore the effects of three main administration parameters, sagittal (head) angle, coronal angle and insertion depth of the nasal spray tip, and their interactions, a Box-Behnken design (i.e., a type of response surface design (RSD)), was utilized. The Box-Behnken experimental design is effective in reducing the number of experimental runs while maintaining a good understanding of the underlying responses and has the ability to provide reliable results and insights for quadratic models [52]. In this design, each administration parameter was evaluated at three levels including lower, middle, and upper, resulting in three distinct values per variable. The lower level is assigned by −1, the middle level by 0, and the upper level by + 1. Table I shows the experimental design, the real value of each level, and the assigned dimensionless values (−1, 0, or 1).

**Table I:** The Box-Behnken experimental design Table showing both dimensionless values and their corresponding real-world dimensions in parentheses

Case	Sagittal Angle (real values °)	Coronal Angle (real values °)	Insertion Depth (real values mm)
1	0 (C: 57°, A: 67°)	1 (C: 24.8°, A: 36°)	0 (C: 3.6 mm, A: 5.4 mm)
**2**	**−1 (C: 47°, A: 57°)**	**0 (C: 19.8°, A: 31°)**	**1 (C: 6 mm, A: 9 mm)**
3	1 (C: 67°, A: 77°)	0 (C: 19.8°, A: 31°)	1 (C: 6 mm, A: 9 mm)
4	0 (C: 57°, A: 67°)	1 (C: 24.8°, A: 36°)	1 (C: 6 mm, A: 9 mm)
5	1 (C: 67°, A: 77°)	1 (C: 24.8°, A: 36°)	0 (C: 3.6 mm, A: 5.4 mm)
6	−1 (C: 47°, A: 57°)	−1 (C: 14.8°, A: 26°)	0 (C: 3.6 mm, A: 5.4 mm)
7	1 (C: 67°, A: 77°)	0 (C: 19.8°, A: 31°)	−1 (C: 1.2 mm, A: 1.8 mm)
8	0 (C: 57°, A: 67°)	−1 (C: 14.8°, A: 26°)	−1 (C: 1.2 mm, A: 1.8 mm)
9	0 (C: 57°, A: 67°)	0 (C: 19.8°, A: 31°)	0 (C: 3.6 mm, A: 5.4 mm)
10	0 (C: 57°, A: 67°)	−1 (C: 14.8°, A: 26°)	1 (C: 6 mm, A: 9 mm)
11	1 (C: 67°, A: 77°)	−1 (C: 14.8°, A: 26°)	0 (C: 3.6 mm, A: 5.4 mm)
12	−1 (C: 47°, A: 57°)	0 (C: 19.8°, A: 31°)	−1 (C: 1.2 mm, A: 1.8 mm)
13	−1 (C: 47°, A: 57°)	1 (C: 24.8°, A: 36°)	0 (C: 3.6 mm, A: 5.4 mm)

It should be noted that case #2 represents the original configuration, with administration parameters identical to those used for the M models in the study by Khadka *et al*. [2]. To accommodate changes in administration parameters, the orientation and positioning of the spray tip holder within the anterior nasal piece were redesigned. The angular adjustments were applied first, followed by a reduction in insertion depth when necessary. Figures S2 and S3 show the stereolithography models of the customized anterior pieces for each case, corresponding to the child and adult M models, respectively. In this study, three units of Nasacort (NC), all from the same lot, were used to actuate the drug into the nasal models.

The factors are labeled for the child model (C) and adult model (A). Note that non-negative dimensionless values for sagittal angle indicate moving the spray tip upward while positive dimensionless values for the coronal angle indicate aiming more toward the nasal septum. **Case 2** is the original case used by Khadka *et al*. [2]

### Experimental Setup

Experimental setup in this study is similar to that of Khadka *et al*. [2], where detailed descriptions are provided. However, the following section, accompanied by Fig. 1B, provides a brief overview of the setup, apparatus, and experimental methodologies employed in this study:

- A breathing simulator (ASL 5000; IngMar Medical, USA) was used in the *in vitro* deposition study to mimic gentle and vigorous sniffing breathing patterns.
- Vereo® automatic actuators (Proveris Scientific Corporation, MA, USA) were used to actuate both Nasacort and Flonase Sensimist nasal sprays during the *in vitro* regional drug deposition study. The top actuator (Vereo® NSx) was used for Nasacort, and the side actuator (Vereo® SSx) was employed for the Flonase Sensimist.
- For each spray, one actuation was performed at the beginning of an inhalation cycle into the child model, while two actuations were administered at the start of two consecutive inhalation cycles into the adult model. This protocol reflects the different recommended dosing regimens for patients 2–12 years old and those over 12 years.
- Following the actuation, the nasal models, sealed with parafilm, were immediately disassembled. Drug deposition in each segment of the nasal models was recovered by rinsing with an appropriate diluent. The volumes of diluent used were 10 mL for the anterior and 5 mL for all other segments.
- The wash solutions obtained were then analyzed using an established high-performance liquid chromatography (HPLC) method for triamcinolone acetonide and fluticasone furoate [1]. However, the flow rate for quantification of fluticasone furoate, the active pharmaceutical ingredient (API) of Flonase Sensimist, was reduced to 1 ml/min from 1.2 ml/min, which in turn increased the retention time to around 12 min.
- To ensure the reliability and reproducibility of the experimental data, a minimum drug recovery threshold was established. Only experimental runs yielding a total recovered dose (comprising both the mass deposited within the nasal model and the mass recovered from the spray tip) greater than 80% of the nominal dose were included in the analysis; any trials falling below this threshold were repeated. For the purpose of quantifying regional deposition, the percentage of the dose in each anatomical zone was normalized by the total mass recovered from within the model.

Although the models are anatomically accurate and the experiments are carried out using realistic *in vivo*-measured parameters for actuation and breathing, the limitations of this study include a lack of mucus coating within the models, as well as a lack of humidity and temperature control.

### Statistical Analysis

A one-way analysis of variance (ANOVA), followed by Tukey’s post-hoc multiple comparison test, was performed to investigate differences in mean drug deposition across nasal regions under different breathing patterns. A significance level of 0.05 was used for all statistical tests. MATLAB R2022a (MathWorks®, USA) was used for the ANOVA and multiple comparison tests (Tukey’s post-hoc). To analyze the results from the administration parameters sensitivity study, a response surface model was utilized. This model was used to explore the relationship between administration parameters (independent variables) and regional drug deposition (dependent responses). Given that the Box-Behnken design supports quadratic modeling, the fitted model included not only the linear terms of the variables, but also their second order (squared terms) and interactions (cross-product terms). Moreover, this analysis enabled multiple response optimization by maximizing the overall desirability function, defined as the geometric mean of the individual desirability functions for each response variable [53]. In this approach, individual desirability functions are constructed as a transformation of each response variable such that its value is 0 when the expected response is below a specified minimum (here, the minimum predicted value), and is 1 when it exceeds a specific maximum (here, the maximum predicted value), assuming the goal is to maximize the response. When the desirability is set to minimize a response, this approach simply maximizes the negative of the expected response [54]. JMP® Pro 17.0.0 (SAS Institute Inc., USA) was used to perform the response surface model fit and optimization. Finally, the variable importance was assessed based on the extent of variance of the outcome attributed to variation of each factor. The independent uniform inputs option was selected in the software, since all of the variables were uncorrelated and uniformly distributed in their range [13, 53]. Both main and total effects of variables were assessed. The main effect is the isolated impact of a variable on the predicted outcome, while the total effect includes both this direct impact and any interactions with other variables.

## Results and Discussion

### Effect of Breathing Pattern on *In Vitro* Intranasal Regional Drug Deposition

**Fig. 2:**
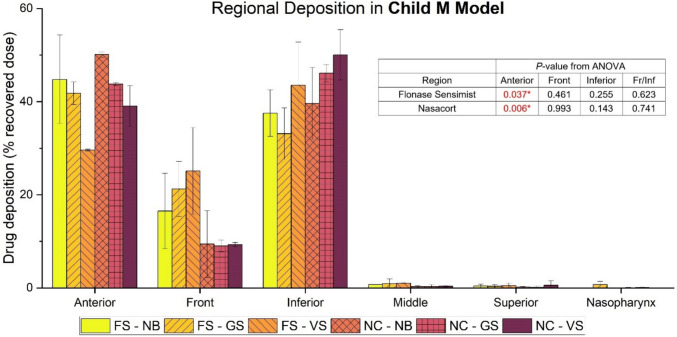
Regional drug deposition in the child model under three different breathing conditions, and the *p*-values obtained from one-way ANOVA comparing the deposition percentages in the three regions with the highest drug deposition (NB = No breathing, GS = Gentle sniffing, VS = Vigorous sniffing, FS = Flonase Sensimist, and NC = Nasacort).

**Fig. 3:**
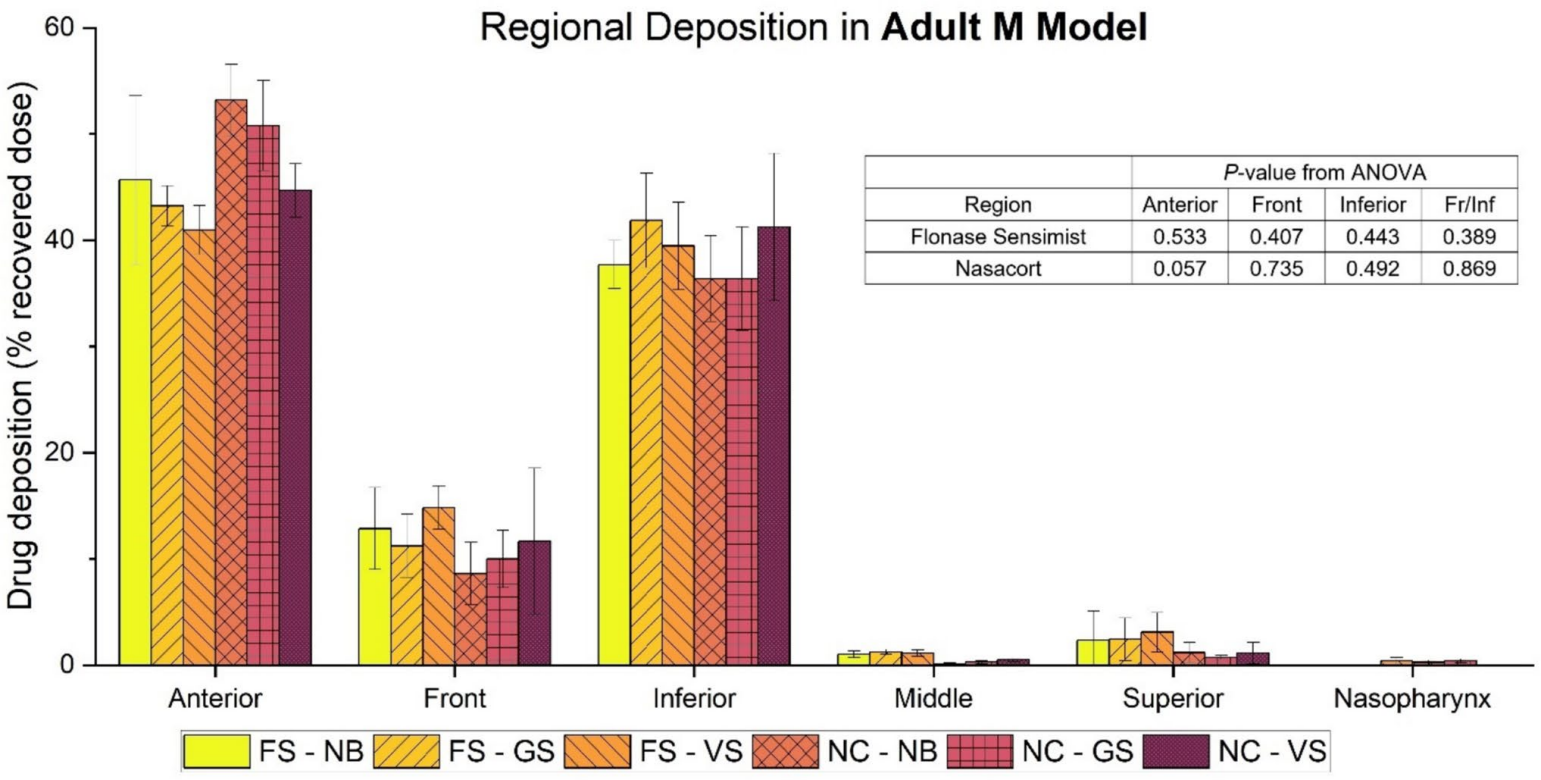
Regional drug deposition in the adult model under three different breathing conditions, and the *p*-values obtained from one-way ANOVA comparing the deposition percentages in the three regions with the highest drug deposition (NB = No breathing, GS = Gentle sniffing, VS = Vigorous sniffing, FS = Flonase Sensimist, and NC = Nasacort).

*In vitro* drug deposition patterns under the three distinct breathing patterns described in Section "Breathing Pattern Sensitivity Analysis" were obtained and compared between the child and adult nasal models using Nasacort and Flonase Sensimist nasal sprays. Figures 2 and 3 show the percentage of recovered drug deposited in each nasal region and demonstrate the transition from no breathing to vigorous sniffing increases posterior drug deposition in both models for both nasal sprays. For both products, the difference between gentle and vigorous sniffing is more pronounced in the child model than in the adult model, particularly for Flonase Sensimist. This could be attributed to the narrower plume of Flonase Sensimist [2], combined with the smaller geometry of the child M model, which facilitates drug delivery to the posterior regions of the nasal cavity during vigorous sniffing. In all cases, more than 95% of the recovered drug is deposited on the anterior, the front, and the inferior turbinate regions. Therefore, these three regions are of particular interest when evaluating the impact of different breathing conditions *in vitro*. In addition to the three aforementioned regions, the ratio of front deposition to inferior turbinate deposition (Fr/Inf) was also analyzed. Since most of the drug deposited in the posterior region ends up in either the inferior turbinate or front (non-turbinate area), this ratio indicates how much of the drug is deposited in the non-turbinate portion of the posterior region [55]. The evaluation was performed using a one-way ANOVA test. Figures 2 and 3 present the *p*-values obtained from the ANOVA for the child and adult models, respectively, under the three breathing conditions for both nasal sprays. A statistically significant difference between breathing patterns was observed only in the anterior region of the child model. Further analysis revealed that this significance arises from the contrast between the no-breathing and vigorous sniff conditions. Gentle breathing, however, did not show a statistically significant difference when compared to either no breathing or vigorous sniffing across any region, spray type, or age group. It should also be noted that the vigorous sniffing pattern employed in this study may represent an unrealistically extreme condition for a 5-year-old child. This finding underscores that, within a range close to gentle breathing, variations in breathing pattern do not result in statistically significant differences in regional drug deposition.

### Impact of Administration Angles and Insertion Depth on Nasal Drug Deposition Patterns

**Fig. 4:**
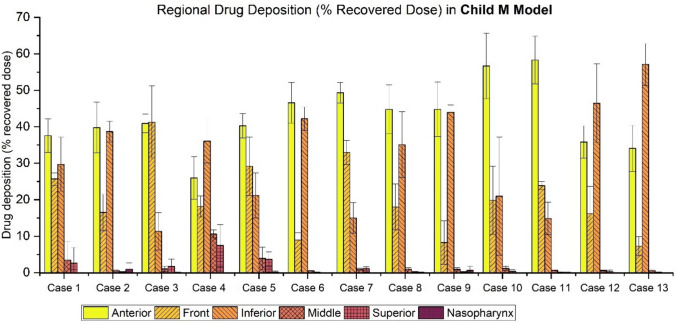
A bar chart illustrating the drug deposition percentage in each region for all 13 cases in the child M model. Each bar represents a specific region, and the different colors/patterns correspond to the individual test cases defined by the Box-Behnken experimental design.

**Fig. 5:**
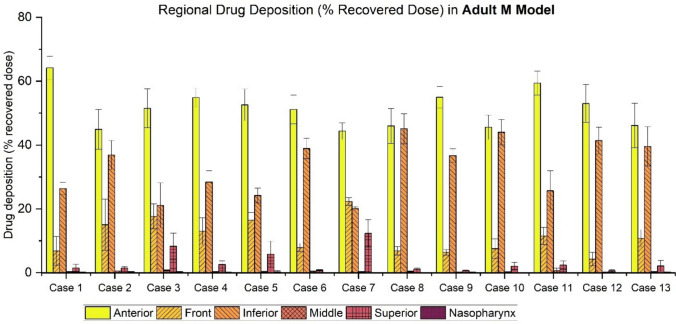
A bar chart illustrating the drug deposition percentage in each region for all 13 cases in the adult M model. Each bar represents a specific region, and the different colors/patterns correspond to the individual test cases defined by the Box-Behnken experimental design.

Using a Box-Behnken experimental design, 13 test cases were developed to evaluate the impact of administration parameters on regional drug delivery (Table I). Compared to the original administration parameters of the nasal models, the insertion depth was reduced (i.e., the spray was pulled further out of the nose), and the sagittal angle was increased (i.e., aimed more upward), to better reflect typical user behavior during nasal spray application, where the coronal angle was both reduced and increased. Figures 4 and 5 summarize the deposition results of all test cases in the child and adult models, respectively.

Prior to conducting statistical analysis, several observations can be made from the results. In the child model, posterior drug deposition ranges from 42 to 74%, with an average of 57%. In contrast, the adult model shows a narrower range, from 36 to 56%, with an average of 49%. The posterior deposition for the original child M model is 60%, aligning closely with the average, while the original adult M model shows a posterior deposition of 55%, which is near the upper bound of the observed range. These findings suggest that, for the same range of variability in administration angles, the child model exhibits greater variation in posterior deposition than the adult model, likely due to its smaller anatomical geometry. Interestingly, in the child model, the original case (Case 2), with patient-specific administration parameters, underperformed in delivering drug to the posterior region compared to Case 4, which achieved the highest posterior deposition. The patient-specific parameters were obtained by projecting the nasal spray plume geometry to minimize interaction with the anterior region, thereby aiming to reduce anterior losses and consequently enhance posterior delivery [10]. This outcome demonstrates how nasal spray flow can propel the drug suspension toward posterior regions even after initial deposition in more anterior areas, as also observed in a recent study by our group [13]. Furthermore, this may help explain why breathing patterns do not significantly influence intranasal drug deposition pattern: the spray-generated flow can dominant the airflow dynamics within the nasal cavity.

Another measurement of interest is the amount of drug recovered from outside of the model, specifically on the tip of the spray, referred to as the dripping amount. In the child model, the highest dripping was observed in Case 10, followed by Cases 11 and 8. For the adult model, Case 11 exhibited the highest dripping, followed by Cases 9 and 10. Notably, most of these cases, particularly Cases 10 and 11, which were common to both models, involved spray orientations directed away from the septum. This suggests that aiming the spray toward the septum may help retain more of the drug within the nasal cavity by potentially reducing external loss.

Finally, it is evident that variations in administration parameters affect regional drug deposition differently in the child and adult M models. While each parameter individually affects regional drug deposition, their impacts on local deposition patterns are not entirely independent. Therefore, statistical analysis is necessary to determine the relative importance of each parameter and the nature of their interactions.

Using dimensionless variables presented in Table I, a linear regression analysis can be performed on the results for the desired region (e.g. anterior) with the response surface effects (i.e., a quadratic model) to optimize the desired outcome. The regression can be done simultaneously across multiple regions, enabling the assignment of distinct desirability criteria for each region. For instance, one may aim to minimize drug deposition in one region (e.g. anterior) and maximize it in another region (e.g. inferior turbinate). It is important to note that non-negative sagittal angle values indicate directing the spray tip upward, whereas positive coronal angle values correspond to orientation toward the nasal septum.

#### Regression Analysis for the Child M Model

As previously mentioned, the study design provides us with considerable flexibility and a variety of options in performing regression analyses, i.e., allowing for performing the regression analysis for any desired region on its own or concurrently in combination with any other desired regions. In this analysis, the whole turbinate region and the anterior were evaluated concurrently, since the goal is to minimize anterior loss while maximizing drug delivery to the entire turbinate region as the target area. The analysis yielded a regression with an *R*^2^ = 0.99 for the anterior and a regression with an *R*^2^ = 0.92 for the turbinate region, indicating strong model fits. The parameter estimates (i.e., the estimated coefficient for each variable) are presented in Table II for both the anterior and the whole turbinate regions.

**Table II:** Parameters estimates (i.e., the estimated coefficient for each variable) for the fitted linear regression model for anterior and turbinate region of the child model, and the condition that has the highest desirability (minimizing anterior deposition while maximizing the turbinate deposition) and the condition that causes the highest turbinate deposition

Anterior			Turbinate		
Term	Estimate	Prob >|t|	Term	Estimate	Prob >|t|
Coronal angle	−8.371	< 0.0001	Sagittal angle	−13.881	0.0167
Coronal × (Insertion-0.07692)	−7.377	0.0008	Coronal × (Insertion-0.07692)	8.913	0.203
Sagittal angle	3.832	0.0008	Coronal angle	6.451	0.1232
Sagittal × (Insertion-0.07692)	−3.094	0.0041	Sagittal^2^	−4.765	0.4153
(Insertion-0.07692)^2^	−2.667	0.0156	(Insertion-0.07692)^2^	−4.668	0.4641
Insertion depth	−1.683	0.0129	Sagittal × (Insertion-0.07692)	1.219	0.7817
Sagittal × Coronal	−1.370	0.0382	Insertion depth	−1.092	0.7616
Coronal^2^	0.677	0.3025	Sagittal × Coronal	−0.468	0.9147
Sagittal^2^	−0.615	0.2945	Coronal^2^	−0.373	0.9518
Intercept	44.762	< 0.0001	Intercept	40.192	0.0127
Maximum Desirability			Maximum Turbinate Deposition		
Coronal angle	1 (47º)		Coronal angle	1 (47º)	
Sagittal angle	−1 (24.8º)		Sagittal angle	−1 (24.8º)	
Insertion depth	1 (6 mm)		Insertion depth	0.781 (5.52 mm)	

Considering all the variables, parameters, and their interaction terms, the absolute value of estimates shows the importance of each variable: the larger the absolute value, the more important the variable on the amount of drug delivered to the region. Thus, for the anterior region, coronal angle is the most important one and its negative value shows that by increasing the coronal angle (going towards the septum), the drug deposition on the anterior region decreases. The most important factor for drug deposition in the turbinate region collectively is the sagittal angle and the negative value shows moving the nasal tip upward decreases the drug deposition in this region, due to increase of drug deposition on the front region. Also, it can be seen that the interactions of the administration parameters generally have a relatively large absolute value, emphasizing the importance of considering the interactions.

**Fig. 6:**
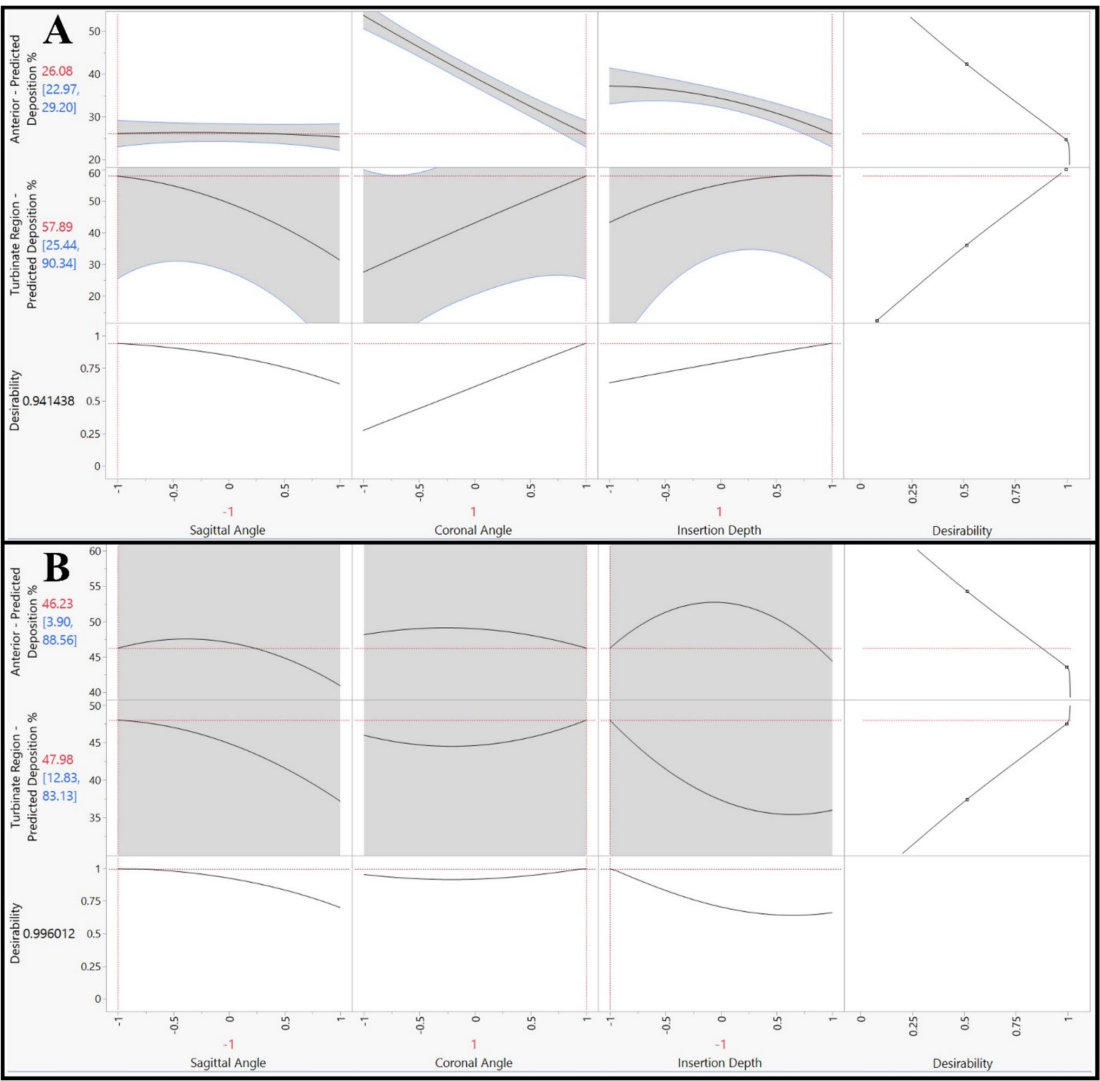
**A**) Prediction and desirability Profilers for anterior and turbinate deposition of the child model. **B**) Prediction and desirability Profilers for anterior and turbinate deposition of the adult model. The gray area represents the 95% confidence interval. The first two rows in each panel show how parameters affect deposition in the anterior and turbinate regions, respectively. The third row illustrates how administration parameters influence the desirability, which was set to minimize anterior and maximize turbinate deposition, reflected in the negative and positive slopes, respectively. Dashed lines intercepting the *x*-axis (and values under the *x*-axis) indicate values corresponding to the maximized desirability (Sagittal = −1, Coronal = Insertion = 1, for the child model as an example). Dashed lines intercepting *y*-axis (and values left to the *y*-axis) show predicted deposition percentage or the value of desirability (26.08% is predicted for anterior deposition of the child model, as an instance).

Using the above regressions, we can find the most favorable condition to have the highest deposition in the turbinate region. Figure 6A shows the Prediction Profiler, a JMP Pro® visualization feature, for anterior and turbinate region. This feature visualizes the effect of main effects (administration parameters) on the response (deposition percentage) one at a time, and also can be used to optimize the response based on the set desirability. In Fig. 6A, the selected point is where the model has predicted that the turbinate deposition will be the highest. This point corresponds to a coronal angle of 24.8º, sagittal angle of 47º and insertion depth of 5.52 mm (Table II). The percentage drug deposition in this situation is predicted to be 58.1%, slightly higher than case 13, which had the highest turbinate drug deposition. The difference here is only the insertion depth, which was 3.6 mm in case 13 and 5.52 mm for the optimal predicted case. However, if the desirability is set at minimizing the anterior deposition while maximizing the turbinate deposition, the maximum desirability is achieved at sagittal angle = −1 (24.8º), coronal angle = 1 (47º), and insertion depth = 1 (6 mm), where the predicted anterior deposition is 26.08% and turbinate deposition is predicted to be 57.89% (Table II).

The values that should be used for variables to multiply by the estimates and sum them to get the predicted deposition are the dimensionless ones (−1 to 1).

An alternative approach to data analysis involves examining one region at a time to optimize deposition within that specific area, or to assess the influence of each variable — either as a main effect or through interactions with other variables — on regional deposition. Table III shows the ranked order of importance of variables for each region. In the child M model, adjusting coronal angle by aiming the spray tip towards septum can significantly reduce the deposition loss at anterior region. However, this contrasts with nasal spray usage guidelines, which typically recommend aiming the spray tip away from the septum to avoid nasal septal perforation associated with prolonged use of nasal sprays.

**Table III:** Ranking of the three administration parameters based on their main and total effect on drug deposition in each region for both of the child and adult models. Asterisk (*) and bolded variables had p-values less

Region	Model	Main Effect	Total Effect
Anterior	Child	Coronal* > Sagittal* > Insertion*	Coronal > Insertion > Sagittal
	Adult	Insertion > Sagittal > Coronal	Insertion > Sagittal > Coronal
Front	Child	Sagittal* > Insertion > Coronal	Sagittal > Insertion > Coronal
	Adult	Sagittal* > Insertion > Coronal	Sagittal > Insertion > Coronal
Inferior Turbinate	Child	Sagittal* > Insertion > Coronal	Sagittal > Insertion > Coronal
	Adult	Sagittal* > Coronal > Insertion	Sagittal > Coronal > Insertion
Middle Turbinate	Child	Coronal > Insertion > Sagittal	Coronal > Insertion > Sagittal
	Adult	Insertion > Sagittal > Coronal	Insertion > Sagittal > Coronal
Superior Turbinate	Child	Coronal > Insertion > Sagittal	Coronal > Insertion > Sagittal
	Adult	Sagittal > Insertion > Coronal	Sagittal > Insertion > Coronal
Turbinate Region	Child	Sagittal* > Coronal* > Insertion	Sagittal > Coronal > Insertion
	Adult	Sagittal > Insertion > Coronal	Sagittal > Insertion > Coronal
Nasopharynx	Child	Coronal > Insertion > Sagittal	Coronal > Insertion > Sagittal
	Adult	Coronal > Insertion > Sagittal	Coronal > Insertion > Sagittal

Figure S4 shows the Prediction Profiler with 95% confidence intervals for all the six regions (i.e., anterior, front, inferior turbinate, middle turbinate, superior turbinate, and nasopharynx), under the condition where all variables are set to zero. It should be noted that these profiles are sensitive to changes in the values of other variables. For instance, the effect of insertion depth may become negligible, and the profile plateaus at certain sagittal or coronal angles. This behavior is consistent across all three variables and in all regions. Figure S5 depicts some examples of this behavior, and Equations S1-S6 are the prediction expressions for the six regions in the child M model. Using these equations, one can optimize the drug deposition in any desired region, superior turbinate for instance. However, it should be noted that optimizing with this approach will not allow us to optimize two or more regions at the same time.

#### Regression Analysis for the Adult M Model

The same regression analysis was performed for the adult model in the anterior and turbinate regions. The fitted second-order models yielded an *R*^2^ value of 0.77 for the turbinate region and 0.59 for the anterior region. Although these regressions do not explain as much variation as for the child M model, they can still offer insights into finding the most important factors influencing the percentage of drug deposition in each region. Similar to the previous section, we can evaluate the relative importance of each term by examining the parameter estimates. Table IV summarizes the parameters estimates for the regression models corresponding to the anterior and turbinate regions.

**Table IV:** Parameters estimates (i.e., the estimated coefficient for each variable) for the fitted linear regression model for anterior and turbinate regions of the adult model, and the condition that yields the highest desirability (minimizing anterior deposition while maximizing the turbinate deposition) and the condition that causes the highest turbinate deposition. The values that should be used for variables to multiply by the estimates and sum them to get the predicted deposition are the dimensionless ones (−1 to 1)

Anterior			Turbinate		
Term	Estimate	Prob >|t|	Term	Estimate	Prob >|t|
(Insertion-0.07692)^2^	−7.395	0.262	Sagittal angle	−5.123	0.111
Sagittal × (Insertion-0.07692)	3.796	0.399	(Insertion-0.07692)^2^	4.655	0.373
(Sagittal angle)^2^	−3.456	0.529	Coronal × (Insertion-0.07692)	−3.332	0.503
Coronal × (Insertion-0.07692)	2.067	0.722	Coronal angle	−2.685	0.349
Sagittal angle	1.869	0.546	(Coronal angle)^2^	2.381	0.636
(Coronal angle)^2^	−1.805	0.763	(Sagittal angle)^2^	−2.290	0.610
Coronal angle	0.828	0.795	Insertion depth	−1.773	0.548
Sagittal × Coronal	−0.444	0.916	Sagittal × (Insertion-0.07692)	0.161	0.963
Insertion depth	−0.328	0.924	Sagittal × Coronal	−0.096	0.978
Intercept	58.469	0.004	Intercept	34.423	0.011
Maximum Desirability			Maximum Turbinate Deposition		
Coronal angle	−1 (26º)		Coronal angle	−1 (26º)	
Sagittal angle	−1 (57º)		Sagittal angle	−1 (57º)	
Insertion depth	1 (9 mm)		Insertion depth	1 (9 mm)	

Although the quadratic model for the anterior region showed a moderate fit (R^2^ = 0.59), a diagnostic evaluation was conducted using Support Vector Regression (SVR) to confirm whether the three chosen administration parameters were sufficient to describe the deposition variance. By utilizing a more flexible functional form, the SVR model achieved a Leave-One-Out Cross-Validation (LOOCV) R^2^ of 0.99 (Code and results presented in section S3 of the supplementary document). This high predictive accuracy on unseen data points indicates that the selected independent variables successfully capture the underlying physical signal, suggesting that the lower R^2^ of the linear model is primarily a result of the high non-linearity of the adult anatomy rather than missing variables. Consequently, the parameter estimates in Table IV are presented as a means to identify the directional trends and relative influence of each factor within this complex design space.

For the anterior region, the most important term is the quadratic effect for insertion depth. Its negative coefficient indicates that by increasing the insertion depth, the drug deposition on this region decreases. The most important factor for the drug deposition in the turbinate region collectively is the sagittal angle. The negative sign of its coefficient suggests that directing the spray upward (i.e., increasing the sagittal angle) reduces drug deposition in this region.

**Fig. 7:**
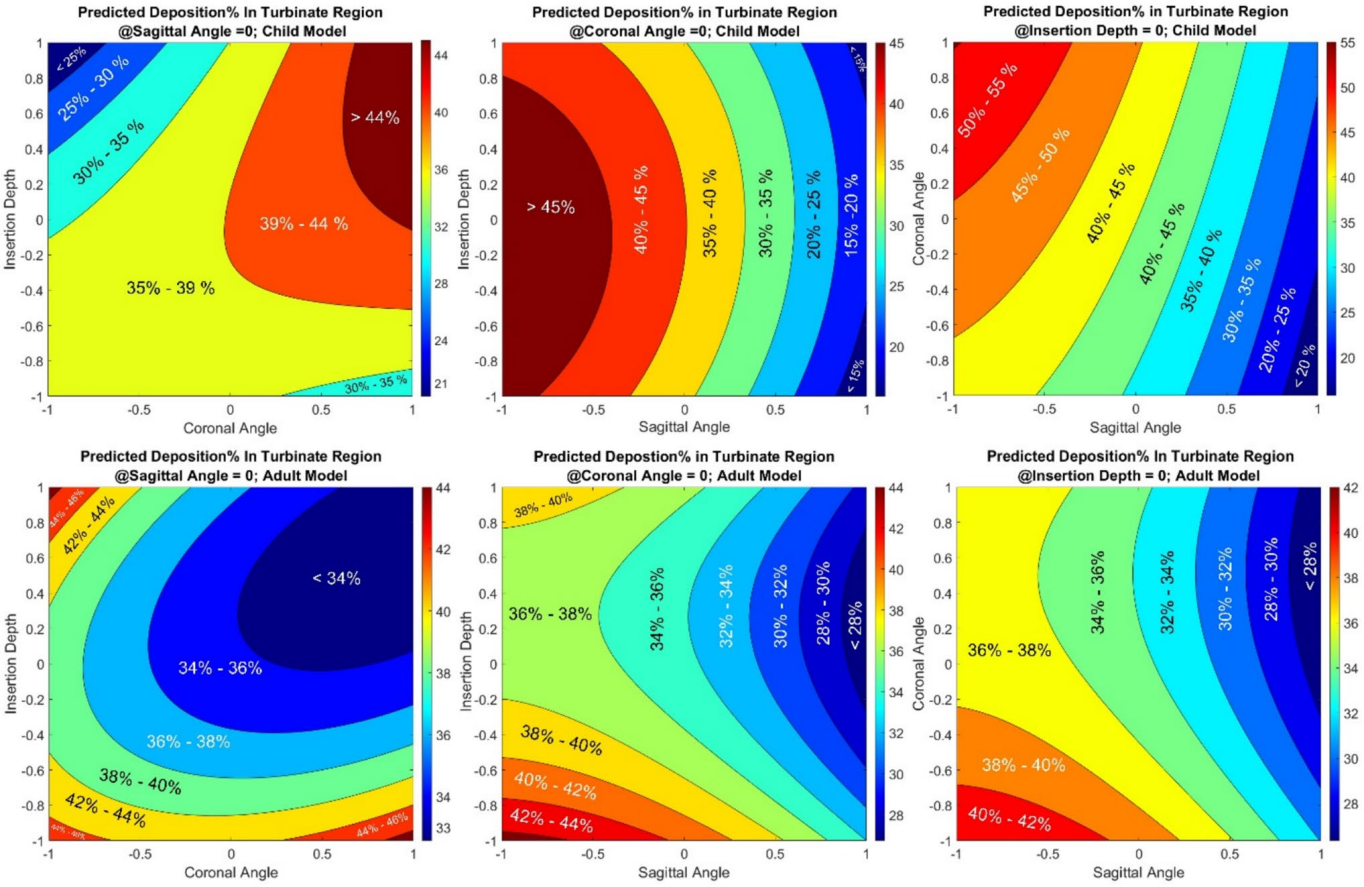
Contours of predicted drug deposition percentage in the turbinate region for both child and adult models, illustrating the effect of varying two administration parameters while keeping the third variable constant at zero, one variable at a time.

Using the fitted models, we identified the optimal conditions that maximize drug deposition in the turbinate region while minimizing deposition in the anterior region. Figure 6B shows the prediction profiles for both regions. The highlighted point represents the condition under which the model predicts the highest turbinate deposition. This point corresponds to a coronal angle of 26º, sagittal angle of 57º, and insertion depth of 9 mm. Under this condition, the predicted drug deposition in the turbinate region is 47.35%, which is slightly higher than that of Case 10— the conditions previously yielding the highest turbinate drug deposition. The difference here is only in the sagittal angle, which was 67º in Case 8 and 57º for the optimal case. Unlike the child model, this optimal set of administration parameters yields the highest predicted deposition in the turbinate region. Another key difference lies in the coronal orientation of the nasal spray when aiming to maximize turbinate deposition. Comparing the results from Tables II and IV shows that aiming towards the septum enhances turbinate deposition in children, whereas to increase deposition in this region in the adult model, the spray should aim away from the septum. Figure 7 further highlights the differences between the child and adult models. As shown, when the sagittal angle is at the mid-level (0), increasing both the insertion depth and sagittal angle, effectively aiming more toward the septum, increases turbinate deposition in the child model. In contrast, the same adjustments result in decreased turbinate deposition in the adult model. Comparisons across other similar cases also demonstrate clear differences between the child and adult models, underscoring the influence of anatomical and airflow differences on deposition patterns.

Figure S6 presents the Prediction Profiler with 95% confidence intervals for all six regions, with all variables set to zero. Equations S7-S12 are the predictive expressions for each of the six regions in the adult M model. Following the same analytical approach used for the child M model, Table III presents the order of importance of the three administration variables in all the regions for the adult M model. A comparison of these rankings between the child and the adult models shows that only for two regions, the front and nasopharynx, the order of variable importance is the same, but the rankings differ in all other regions. This highlights how identical changes made to usage techniques can lead to markedly different deposition outcomes between the child and adult models.

#### Summary of the Statistical Findings from the Administration Parameter Sensitivity Analysis

For the child model:

- It was observed that to decrease the anterior deposition, aiming towards the nasal septum is significantly effective (coronal angle parameter estimate is −8.4). If this is accompanied by increasing the insertion depth, the effect can be intensified (coronal*(insertion-0.077) parameter estimate is −7.4).
- Then, to increase the chance of drug to be deposited in the turbinate region, especially the inferior turbinate, not aiming upward is a key factor (sagittal angle parameter estimate is −13.9). This decreases the percentage drug deposition of the front region of the posterior.
- To maximize the deposition in the superior turbinate, where the olfactory region is located, the spray tip should be fully inserted, aiming upward and toward the septum. The predicted deposition in the superior turbinate for insertion depth = sagittal angle = coronal angle = 1 is 7.58%.

For the adult model:

- None of the three parameters (i.e., insertion depth, sagittal and coronal angles) were found to be significantly important directly. However, the model identifies a localized trend regarding insertion depth, where a mid-level depth (approximately 5.4 mm) was found to increase drug deposition on the anterior region ((insertion-0.077) *(insertion-0.077) parameter estimate is −7.4).
- Predictive trends from the model suggest that, to increase the chance of drug delivery to the turbinate region, similar to the child model, sagittal angle is important and not aiming upward (between 0 and 1, refer to Table I for real values) helps the drug to reach the turbinate region, especially the inferior turbinate.
- Based on the response surface model predictions, to maximize the deposition in the superior turbinate, spray should be aimed upward and toward the septum, but insertion depth should be low. The predicted deposition in the superior turbinate for coronal angle = sagittal angle = 1 and insertion depth = −1 is 13.09%.

The results of the study by Warnken *et al*. [14] align with our findings for coronal and sagittal angles. They found the optimal sagittal angle for increasing posterior deposition in both children and adult subjects to be around 30°, indicating a preference for not aiming upward. More interestingly, they observed that the optimal coronal angle for children is oriented more toward the septum than in the adult models, which is similar to our conclusion. Regarding the insertion depth, Kundoor and Dalby [56] showed that this parameter is of less importance compared to the administration angles, a finding corroborated by Esmaeili *et al*. [57]. These conclusions are consistent with our findings. As shown in Fig. 7, we can observe that insertion depth, on its own, does not govern turbinate deposition as strongly as the two administration angles; rather, its impact is modulated by its interaction with those angles.

#### Limitations

A noted limitation of this study is that the anatomical response appears to follow a complex curvature that exceeds the geometric flexibility of a standard quadratic model in the adult model. Furthermore, the sample size (*N* = 13) limits the statistical power of the ANOVA to identify individual significant predictors at the 0.05 level for certain regions. Despite these constraints, the high predictive accuracy of the non-linear diagnostic model validates the choice of independent variables and suggests that the linear model remains a useful, albeit simplified, representation of the administration parameter effects. In addition to these statistical considerations, the physical models lack a mucus coating as well as humidity and temperature control. The latter likely does not impact the results since droplets exit nasal sprays with high velocity, resulting in low residence time. This implies that humidity and temperature would not have enough time to exert any hygroscopic effects on the droplets and, subsequently, on the deposition patterns. However, the effect of mucus could be meaningful, as is suggested by previous studies [58–60], and warrants further investigation.

## Conclusions

*In vitro* regional drug deposition in the nasal cavity of a child and adult anatomically segmented model was investigated under different breathing and administration conditions. It was shown that breathing pattern does not significantly affect the drug deposition pattern in the adult model, even with consideration of two extremes, no breathing and vigorous sniffing. However, in the child model, a significant difference was observed in the anterior deposition between the vigorous sniffing and no breathing, while the gentle sniffing was not found to be significantly different from the two extremes in any region. The small differences in intranasal deposition pattern using a nasal spray might be attributed to the dominance of the spray flow over the breathing flow. The sprays reach velocities of 10–12 m/s within 100 ms. At that point, the average airflow velocity at the INV plane is around 2.6 m/s in the child model and 1.7 m/s in the adult model within 100 ms of vigorous sniffing. Based on Table S1, the flow rate at 100 ms is approximately 0.199 L/sec, with cross-sectional areas at the INV plane of 118.64 mm^2^ for the adult model [12] and 75.48 mm^2^ for the child model [13].

In contrast to breathing pattern, changes in the administration parameters (variability in usage techniques), captured across the 13 Box-Behnken design cases, resulted in a wide range of drug deposition percentages in the posterior regions of both child and adult models (42% to 74% for the child, and 36% to 56% for the adult models). More importantly, the patterns of drug deposition varied distinctly between the child and adult models, meaning that altering an administration parameter can lead to opposite effects in the same anatomical region across the two models (i.e., changing an administration parameter can increase the deposition in a region of the child nasal cavity while decrease it in the same region of the adult nasal cavity). For example, increasing the coronal angle (i.e., aiming the spray tip toward the septum) enhances drug delivery to the turbinate region in the child model, whereas it decreases the turbinate deposition in the adult model. These findings underscore the importance of considering the influence of administration parameters on *in vitro* regional drug delivery in both pediatric and adult population for *in vitro *assessments. While using only one subject per age group limits generalizability, our study demonstrates that response surface methodology can effectively capture the intrinsic relationships between administration parameters and regional deposition patterns.

Taken together, this work presents a case example of the utility of using the developed pediatric and adult nasal models to better understand how complex dose administration factors can affect drug delivery *in vitro*. Through the use of two locally acting nasal spray drug products, the *in vitro* deposition data and developed modeling approaches showed how* in vitro* nasal drug delivery can be optimized through identifying optimal administration conditions tailored to the therapeutic goals of a given nasal product, based on the desired regional deposition profile. The approaches developed here could serve as useful tools for nasal spray product development investigating the performance of a nasal spray product that is meant to be used in both adult and pediatric patient populations. Additionally, this study offers valuable insights into targeted intranasal drug delivery. Future work may center on the ability of these anatomical nasal models to function across different classes of drug products including drug products that utilize a nasal spray for nose-to-brain delivery, where targeting drug delivery to the region of interest within the nasal cavity may prove to be a crucial step for achieving adequate therapeutic effects.

## Supplementary Information

Below is the link to the electronic supplementary material.

ESM 1 (DOCX 2.44 MB)

ESM 2 (XLSX 27.4 KB)

ESM 3 (TXT 4.23 KB)
