# Supplementary material for: Regional Nasal Drug Deposition in Pediatric vs. Adult Models: In vitro Insights into User Technique and Breathing Patterns Sensitivity
Source: Pharm Res. 2026 Mar 16;43(4):1141–56. doi: 10.1007/s11095-026-04066-8 (PMC13092986; doi:10.1007/s11095-026-04066-8)
Supplement: Supplementary file 1 — (DOCX 2.44 MB) [file 11095_2026_4066_MOESM1_ESM.docx]

**Regional Nasal Drug Deposition in Pediatric vs. Adult Models: In vitro Insights into User Technique and Breathing Patterns Sensitivity**

Mohammad Hejazi^1^, Xiomara Owen^1^, David J. Edwards^2^, Michael Hindle^3^, Worth Longest^1,3^, Theodore Schuman^4^, Ross Walenga^5^, Steven Chopski ^5^, Anubhav Kaviratna^6^, Bryan Newman^6^, and Laleh Golshahi^1,7,*^

^1^ Department of Mechanical and Nuclear Engineering, Virginia Commonwealth University, Richmond, VA, USA

^2^ Department of Statistical Sciences and Operations Research, Virginia Commonwealth University, Richmond, VA, USA. Current address: Department of Mathematical Sciences, The Citadel, Charleston, SC, USA

^3^ Department of Pharmaceutics, Virginia Commonwealth University, Richmond, VA, USA

^4^ Department of Otolaryngology - Head and Neck Surgery, VCU Health, Richmond, VA, USA

^5^ Division of Quantitative Methods and Modeling, Office of Research and Standards, Office of Generic Drugs, Center for Drug Evaluation and Research, U.S. Food and Drug Administration, Silver Spring, MD, USA

^6^ Division of Therapeutic Performance I, Office of Research and Standards, Office of Generic Drugs, Center for Drug Evaluation and Research, U.S. Food and Drug Administration, Silver Spring, MD, USA

^7^ Department of Biomedical Engineering, Virginia Commonwealth University, Richmond, VA, USA

*Corresponding Author: Laleh Golshahi, Ph.D., Room 4326, 401 W Main St, Richmond, VA, 23220, e-mail: lgolshahi@vcu.edu, Phone: +1 (804) 827-3742

**S1. Breathing Pattern Study**

**Table S1.** Details of the breathing conditions from the study by Guo et al. [1] used to study the effect of breathing patterns on regional nasal drug deposition. [1]

| **Breathing pattern** | **Tidal volume (mL)** | **PFR (L/min)** | **T_max_ (s)** | **T_d_ (s)** | **Slope (L/min s^−1^)** |
| --- | --- | --- | --- | --- | --- |
| Slow (gentle) | 560.6 ± 260.3 | 20.2 ± 8.0 | 0.4 ± 0.5 | 1.9 ± 1.0 | 50.9 ± 29.9 |
| Fast (vigorous) | 619.1 ± 517.7 | 35.8 ± 14.1 | 0.3 ± 0.4 | 1.2 ± 0.6 | 133.6 ± 61.3 |


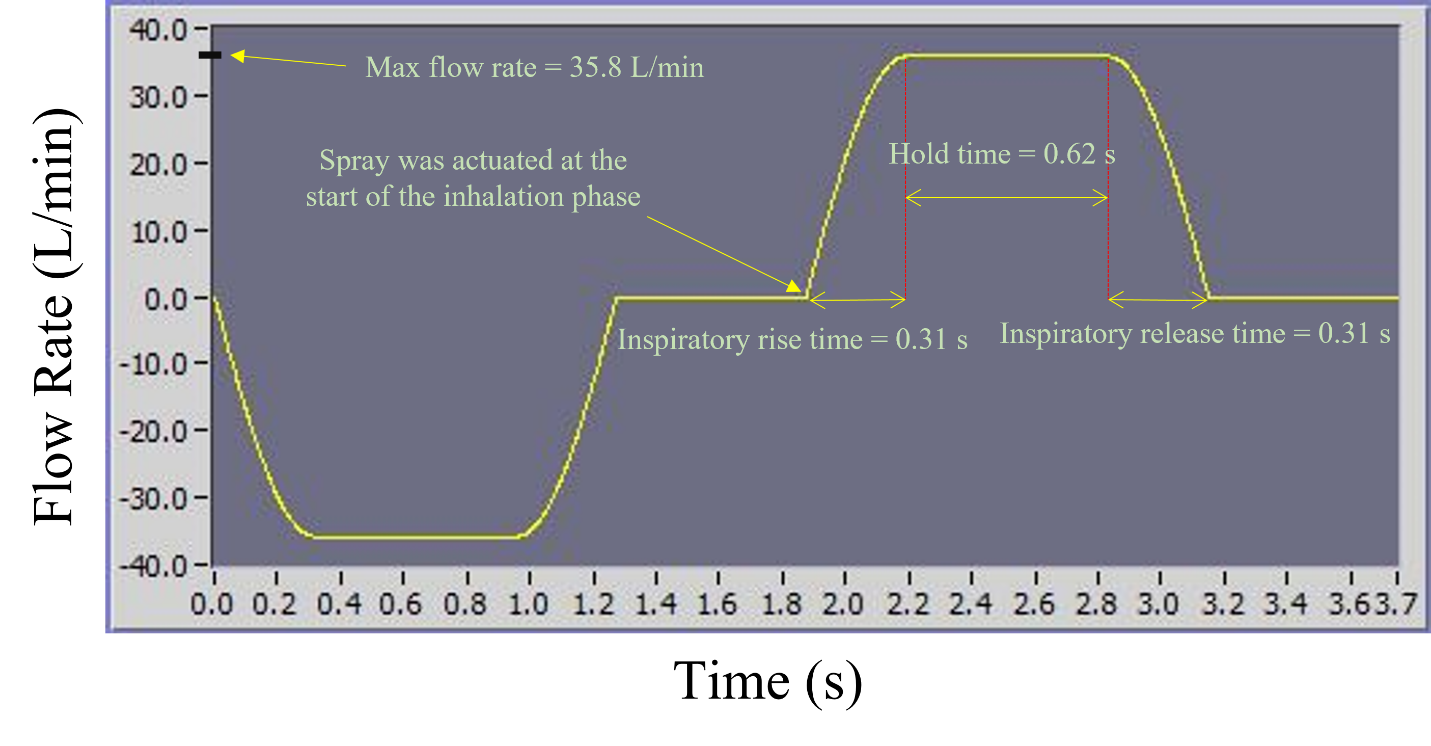


**Figure S1.** Vigorous sniffing breathing profile. Gentle sniffing profile is available in supplementary information in the study by Esmaeili et al. study [2] and Table S1.

**S2. Administration Parameters Sensitivity Study**

**S2.1. Study Design and Spray Tip Holder Designs**


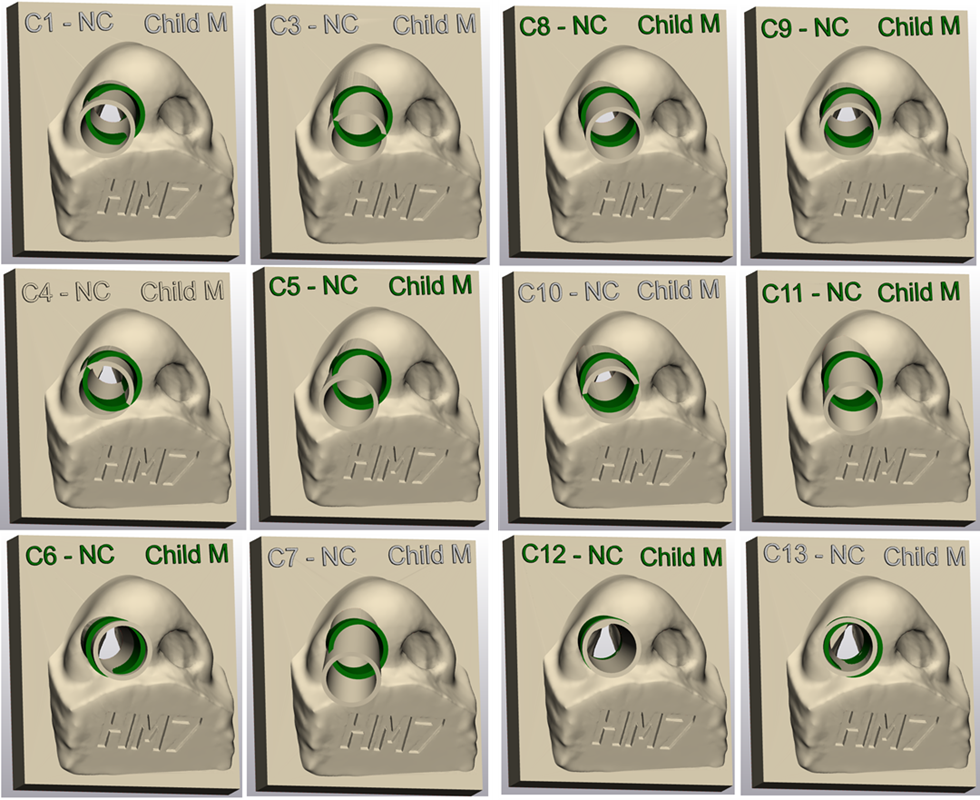


**Figure S2.** Anterior parts designed for Box-Behnken cases of the child M model. The green tip holder represents the original configuration, corresponding to Case #2.


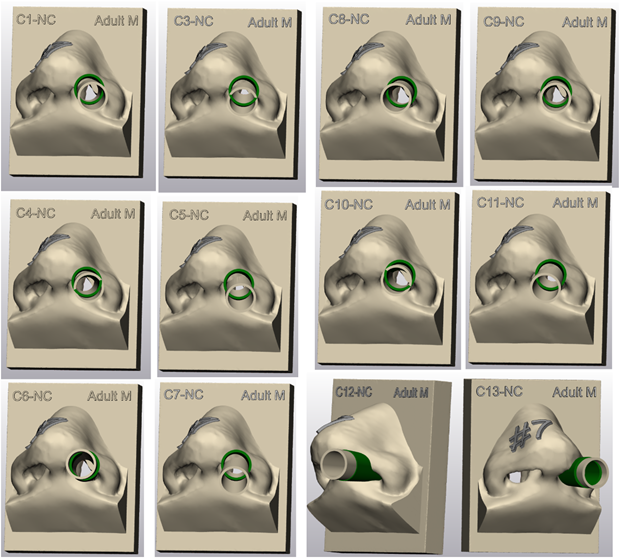


**Figure S3.** Anterior parts designed for Box-Behnken cases of the adult M model. The green tip holder represents the original configuration, corresponding to Case #2.

**S2.2. Sensitivity Study Results**


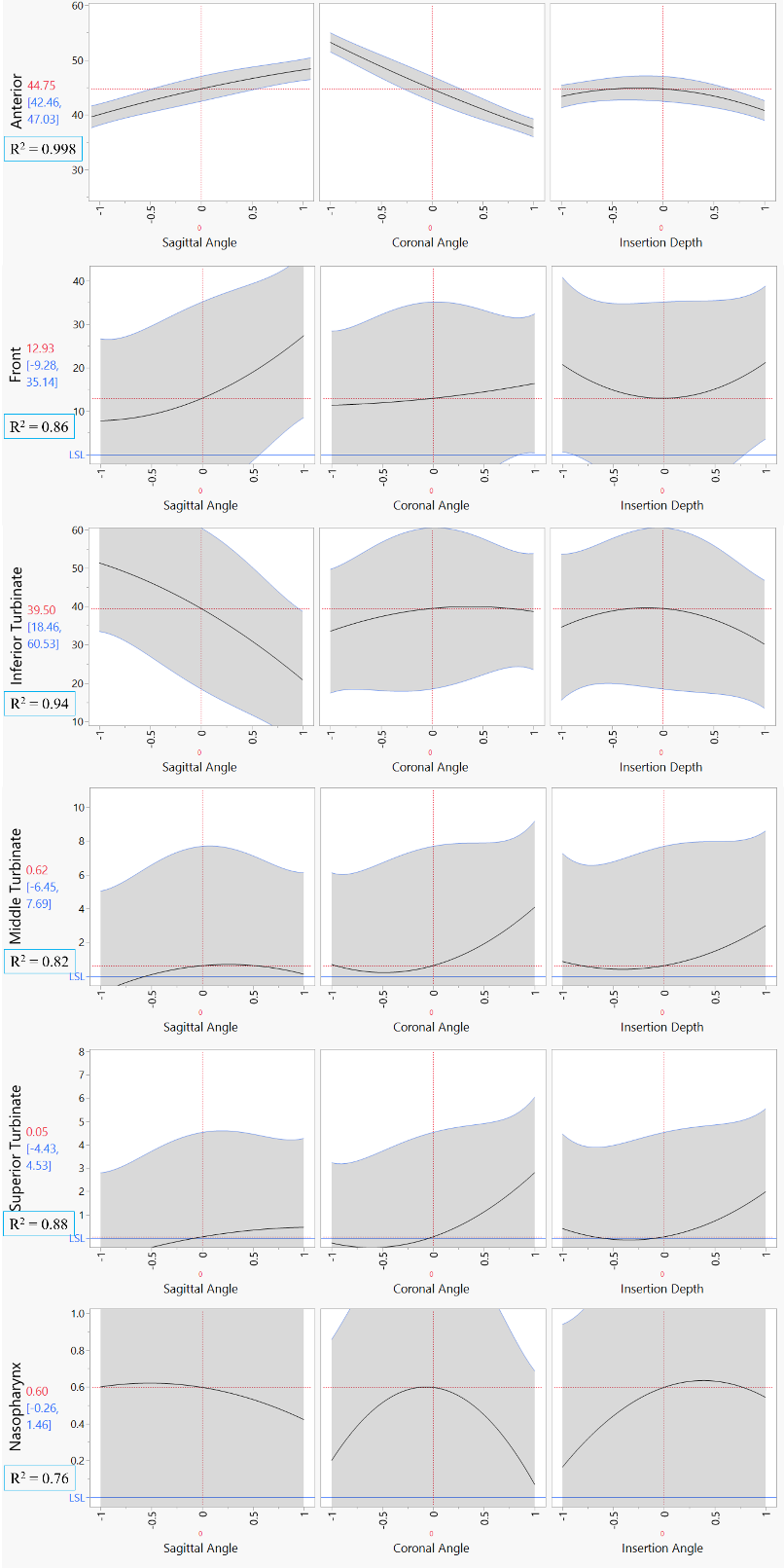


**Figure S4.** The Prediction Profile for the different regions of the nasal cavity in the child model, showing the predicted deposition (red dashed lines intercepting y-axis, 44.75% for anterior as an example) where all the variables were set at 0 (red dashed lines intercepting x-axis). The gray area represents the 95% confidence interval.


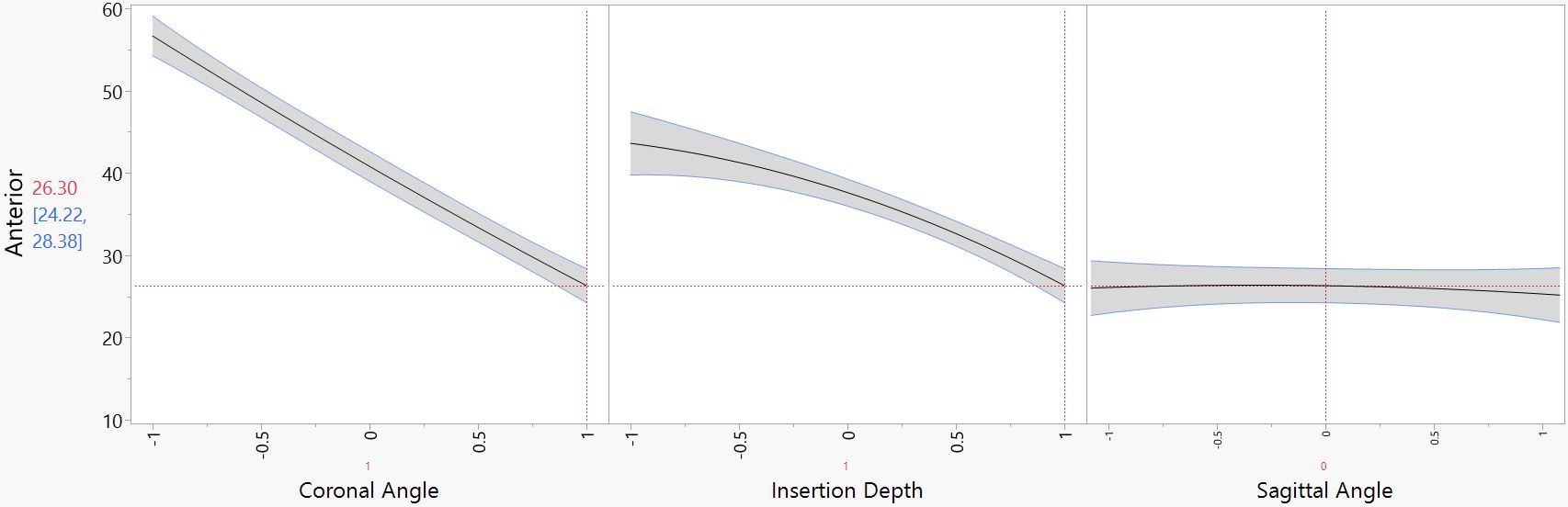


**Figure S5.** The Prediction Profiler for the anterior of the child model when the coronal angle and insertion depth were set to their maximum values (max value = 1) shows that the deposition (y-axis) in this region becomes almost independent of the sagittal angle.

Predictive models for each region of the child model. Note that dimensionless variables have to be implemented into the equations. To convert real (dimensioned) variables into dimensionless form and vice versa, linear interpolation can be applied using reference values from Table I. For example, in the child model, -1 corresponds to a sagittal angle of 47‌°, and 0 corresponds to 57°. Therefore, using linear interpolation, a real angle of 50° maps to a dimensionless value of -0.7.

| $Anterior deposition\%= (44.762)+(3.832\times sagittal angle)+(-8.371\times coronal angle)+(-1.683\times insertion depth)+(sagittal angle\times(sagittal angle\times-0.615))+(sagittal angle\times(coronal angle\times-1.370))+(coronal angle\times(coronal angle\times0.677))+(sagittal angle\times((insertion depth-0.077)\times-3.094))+(coronal angle\times((insertion depth-0.077)\times-7.377))+((insertion depth-0.077)\times((insertion depth-0.077)\times-2.667))$ | Equation S1 |
| --- | --- |
| $Front deposition\%= (12.881)+(9.957\times sagittal angle)+(2.301\times coronal angle)+(1.466\times insertion depth)+(sagittal angle\times(sagittal angle\times4.636))+(sagittal angle\times(coronal angle\times1.756))+(coronal angle\times(coronal angle\times0.916))+(sagittal angle\times((insertion depth-0.077)\times1.988))+(coronal angle\times((insertion depth-0.077)\times-2.593))+((insertion depth-0.077)\times((insertion depth-0.077)\times8.003))$ | Equation S2 |
| $Inferior trubinate deposition\%= (39.538)+(-15.177\times sagittal angle)+(2.985\times coronal angle)+(-3.316\times insertion depth)+(sagittal angle\times(sagittal angle\times-3.389))+(sagittal angle\times(coronal angle\times-2.156))+(coronal angle\times(coronal angle\times-3.381))+(sagittal angle\times((insertion depth-0.077)\times1.036))+(coronal angle\times((insertion depth-0.077)\times5.479))+((insertion depth-0.077)\times((insertion depth-0.077)\times-7.115))$ | Equation S3 |
| $Middle turbinate deposition\%=(0.611)+(0.546\times sagittal angle)+(1.850\times coronal angle)+(1.259\times insertion depth)+(sagittal angle\times(sagittal angle\times-1.048))+(sagittal angle\times(coronal angle\times0.805))+(coronal angle\times(coronal angle\times1.763))+(sagittal angle\times((insertion depth-0.077)\times0.020))+(coronal angle\times((insertion depth-0.077)\times2.041))+((insertion depth-0.077)\times((insertion depth-0.077)\times1.304))$ | Equation S4 |
| $Superior turbinate deposition\%= (0.043)+(0.750\times sagittal angle)+(1.616\times coronal angle)+(0.964\times insertion depth)+(sagittal angle\times(sagittal angle\times-0.328))+(sagittal angle\times(coronal angle\times0.882))+(coronal angle\times(coronal angle\times1.246))+(sagittal angle\times((insertion depth-0.077)\times0.163))+(coronal angle\times((insertion depth-0.077)\times1.392))+((insertion depth-0.077)\times((insertion depth-0.077)\times1.144))$ | Equation S5 |
| $Nasopharunx deposition\%= (0.599)+(-0.107\times sagittal angle)+(-0.053\times coronal angle)+(0.152\times insertion depth)+(sagittal angle\times(sagittal angle\times-0.086))+(sagittal angle\times(coronal angle\times0.025))+(coronal angle\times(coronal angle\times-0.464))+(sagittal angle\times((insertion depth-0.077)\times-0.230))+(coronal angle\times((insertion depth-0.077)\times0.156))+((insertion depth-0.077)\times((insertion depth-0.077)\times-0.244))$ | Equation S6 |


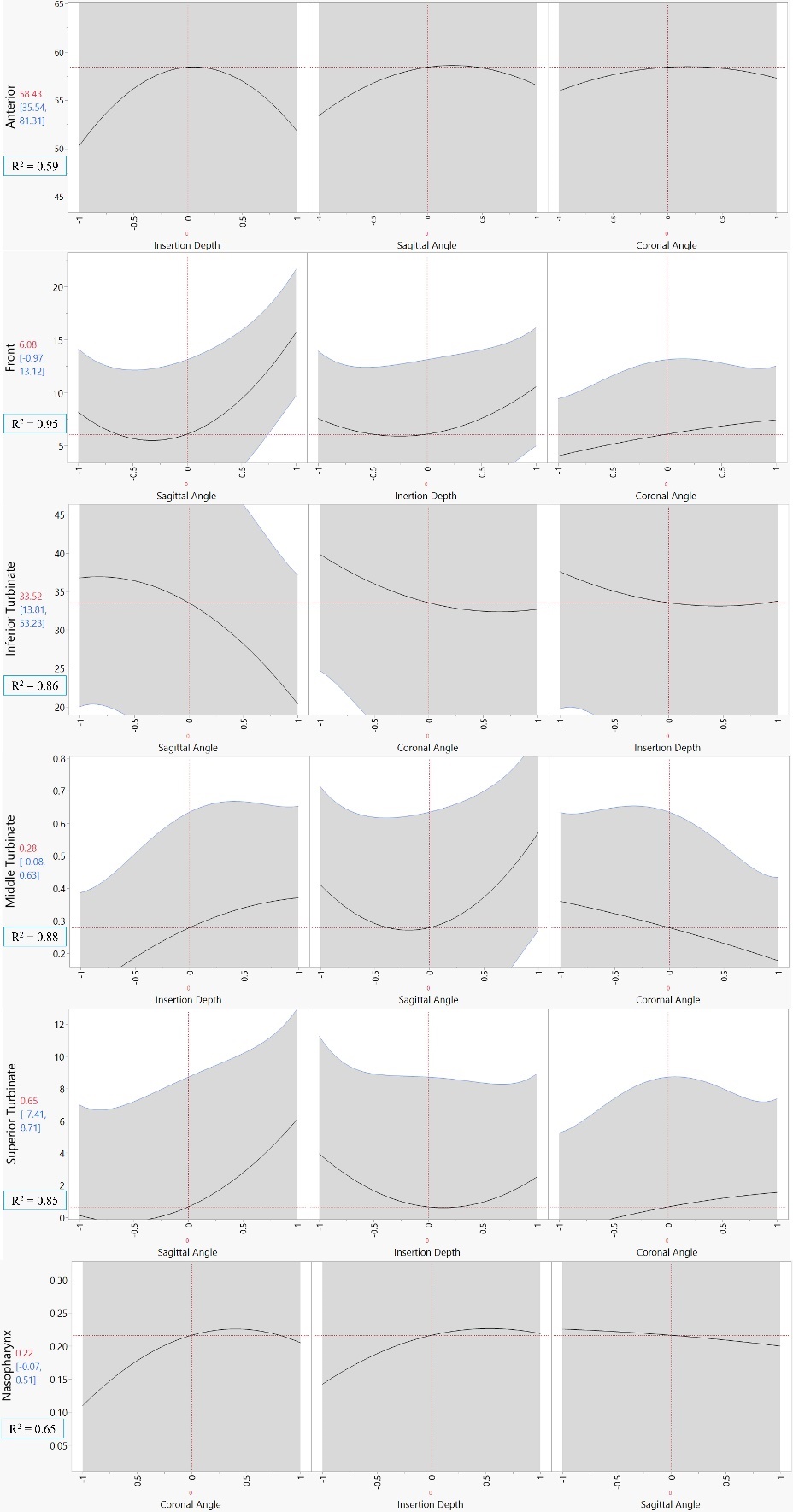


**Figure S6.** Prediction profilers for the different regions of the nasal cavity in the adult model, showing the predicted deposition (red dashed lines intercepting y-axis, anterior deposition is predicted to be 58.43% as an instance) where all the variables were set at 0 (red dashed lines intercepting x-axis). The gray area represents the 95% confidence interval.

Predictive models for each region of the adult model. Note that dimensionless variables have to be implemented into the equations. To convert real (dimensioned) variables into dimensionless form and vice versa, linear interpolation can be applied using reference values from Table I. For example, in the adult model, a coronal angle of 31‌° corresponds to 0, and 36° corresponds to 1. Therefore, using linear interpolation, the dimensionless value for a real angle of 35° is 0.8.

| $Anterior deposition\%= (58.469)+(1.869\times sagittal angle)+(0.828\times coronal angle)+(-0.328\times insertion depth)+(sagittal angle\times(sagittal angle\times-3.456))+(sagittal angle\times(coronal angle\times-0.444))+(coronal angle\times(coronal angle\times-1.805))+(sagittal angle\times((insertion depth-0.077)\times3.796))+(coronal angle\times((insertion depth-0.077)\times2.067))+((insertion depth-0.077)\times((insertion depth-0.077)\times-7.395))$ | Equation S7 |
| --- | --- |
| $Front deposition\%= (6.057)+(3.461\times sagittal angle)+(1.792\times coronal angle)+(1.956\times insertion depth)+(sagittal angle\times(sagittal angle\times5.847))+(sagittal angle\times(coronal angle\times0.523))+(coronal angle\times(coronal angle\times-0.350))+(sagittal angle\times((insertion depth-0.077)\times-3.859))+(coronal angle\times((insertion depth-0.077)\times1.133))+((insertion depth-0.077)\times((insertion depth-0.077)\times2.976))$ | Equation S8 |
| $Inferior trubinate deposition\%= (33.508)+(-8.091\times sagittal angle)+(-3.767\times coronal angle)+(-1.600\times insertion depth)+(sagittal angle\times(sagittal angle\times-4.962))+(sagittal angle\times(coronal angle\times-0.547))+(coronal angle\times(coronal angle\times2.736))+(sagittal angle\times((insertion depth-0.077)\times1.365))+(coronal angle\times((insertion depth-0.077)\times-2.418))+((insertion depth-0.077)\times((insertion depth-0.077)\times2.130))$ | Equation S9 |
| $Middle turbinate deposition\%=(0.279)+(0.082\times sagittal angle)+(-0.081\times coronal angle)+(0.143\times insertion depth)+(sagittal angle\times(sagittal angle\times0.212))+(sagittal angle\times(coronal angle\times-0.071))+(coronal angle\times(coronal angle\times-0.010))+(sagittal angle\times((insertion depth-0.077)\times0.025))+(coronal angle\times((insertion depth-0.077)\times0.137))+((insertion depth-0.077)\times((insertion depth-0.077)\times-0.061))$ | Equation S10 |
| $Superior turbinate deposition\%= (0.636)+(2.886\times sagittal angle)+(1.163\times coronal angle)+(-0.316\times insertion depth)+(sagittal angle\times(sagittal angle\times2.460))+(sagittal angle\times(coronal angle\times0.522))+(coronal angle\times(coronal angle\times-0.345))+(sagittal angle\times((insertion depth-0.077)\times-1.229))+(coronal angle\times((insertion depth-0.077)\times-1.051))+((insertion depth-0.077)\times((insertion depth-0.077)\times2.587))$ | Equation S11 |
| $Nasopharunx deposition\%=(0.216)+(-0.015\times sagittal angle)+(0.048\times coronal angle)+(0.033\times insertion depth)+(sagittal angle\times(sagittal angle\times-0.003))+(sagittal angle\times(coronal angle\times0.047))+(coronal angle\times(coronal angle\times-0.059))+(sagittal angle\times((insertion depth-0.077)\times-0.024))+(coronal angle\times((insertion depth-0.077)\times0.006))+((insertion depth-0.077)\times((insertion depth-0.077)\times-0.036))$ | Equation S12 |

**S3. Support Vector Regression (SVR) Python Code and Results**

import pandas as pd

import numpy as np

from sklearn.svm import SVR

from sklearn.model_selection import GridSearchCV, LeaveOneOut

from sklearn.preprocessing import StandardScaler

from sklearn.metrics import r2_score, mean_squared_error

# 1. Data Setup (Populated from your image and previous text)

data = {

    'sag': [0, -1, 1, 0, 1, -1, 1, 0, 0, 0, 1, -1, -1],

    'cor': [1, 0, 0, 1, 1, -1, 0, -1, 0, -1, -1, 0, 1],

    'ins': [0, 1, 1, 1, 0, 0, -1, -1, 0, 1, 0, -1, 0],

    'Ant': [64.17, 44.91, 51.46, 54.88, 52.57, 51.15, 44.36, 45.98, 54.98, 45.58, 59.39, 53.00, 46.11]

}

df = pd.DataFrame(data)

X = df[['sag', 'cor', 'ins']]

y = df['Ant']

# 2. Scaling (Important for SVR kernels)

scaler_X = StandardScaler()

scaler_y = StandardScaler()

X_scaled = scaler_X.fit_transform(X)

y_scaled = scaler_y.fit_transform(y.values.reshape(-1, 1)).flatten()

# 3. LOOCV Grid Search

loo = LeaveOneOut()

svr = SVR(kernel='rbf')

# Broad search

param_grid = {

    'C': [10, 20, 30, 40, 50, 60, 70, 80, 90],

    'gamma': [0.1, 0.2, 0.3, 0.4, 0.5],

    'epsilon': [0.001, 0.01, 0.1]

}

grid_search = GridSearchCV(

    estimator=svr,

    param_grid=param_grid,

    cv=loo,

    scoring='neg_mean_squared_error'

)

grid_search.fit(X_scaled, y_scaled)

# 4. Results Processing

best_model = grid_search.best_estimator_

y_pred_scaled = best_model.predict(X_scaled)

y_pred = scaler_y.inverse_transform(y_pred_scaled.reshape(-1, 1)).flatten()

# Final Metrics

r2 = r2_score(y, y_pred)

rmse = np.sqrt(mean_squared_error(y, y_pred))

print("--- MODEL SUMMARY ---")

print(f"Best C: {grid_search.best_params_['C']:.2f}")

print(f"Best Gamma: {grid_search.best_params_['gamma']:.4f}")

print(f"Best Epsilon: {grid_search.best_params_['epsilon']}")

print(f"LOOCV R^2: {r2:.4f}")

print(f"LOOCV RMSE: {rmse:.4f}")

# Display Predictions vs Actuals

results_df = pd.DataFrame({'Actual': y, 'Predicted': y_pred, 'Residual': y - y_pred})

print("\n--- PREDICTIONS ---")

print(results_df)

--- MODEL SUMMARY ---

Best C: 10.00

Best Gamma: 0.5000

Best Epsilon: 0.1

LOOCV R^2: 0.9914

LOOCV RMSE: 0.5383

--- PREDICTIONS ---

Actual Predicted Residual

0 64.17 63.588694 0.581306

1 44.91 45.488924 -0.578924

2 51.46 51.292744 0.167256

3 54.88 54.721349 0.158651

4 52.57 53.150576 -0.580576

5 51.15 50.570226 0.579774

6 44.36 44.942915 -0.582915

7 45.98 46.560654 -0.580654

8 54.98 55.562017 -0.582017

9 45.58 46.159666 -0.579666

10 59.39 58.806003 0.583997

11 53.00 52.420754 0.579246

12 46.11 46.692393 -0.582393

**References**

1. Guo Y, Laube B, Dalby R. The effect of formulation variables and breathing patterns on the site of nasal deposition in an anatomically correct model. Pharm Res [Internet]. 2005 [cited 2025 Feb 7];22:1871–8. Available from: https://link.springer.com/article/10.1007/s11095-005-7391-9

2. Esmaeili AR, Wilkins J V., Hosseini S, Alfaifi A, Hejazi M, Hindle M, et al. In vitro evaluation of intersubject variability in pediatric intranasal drug delivery using nasal spray suspension products. J Aerosol Sci. 2024;179:106387.
